# A Surgeon That Switched to Unrestricted Kinematic Alignment with Manual Instruments Has a Short Learning Curve and Comparable Resection Accuracy and Outcomes to Those of an Experienced Surgeon

**DOI:** 10.3390/jpm12071152

**Published:** 2022-07-16

**Authors:** Alexander J. Nedopil, Anand Dhaliwal, Stephen M. Howell, Maury L. Hull

**Affiliations:** 1 Orthopädische Klinik König-Ludwig-Haus, Lehrstuhl für Orthopädie der Universität, 97074 Würzburg, Germany; 2 College of Medicine, California Northstate University, Elk Grove, CA 95757, USA; anand.dhaliwal7125@cnsu.edu; 3 Department of Biomedical Engineering, University of California, Davis, CA 95616, USA; sebhowell@mac.com (S.M.H.); mlhull@ucdavis.edu (M.L.H.); 4 Department of Orthopedic Surgery, University of California, Davis, CA 95616, USA

**Keywords:** total knee arthroplasty, kinematic alignment, learning curve, accuracy, efficiency

## Abstract

After starting an orthopedic practice, a surgeon with a fellowship in mechanically aligned (MA) TKA initiated this study to characterize their learning curve after they switched to unrestricted kinematic alignment (KA) TKA using manual instruments. Accordingly, the present study determined for the inexperienced (IE) surgeon the number of cases required to achieve consistent femoral resections and operating times, and whether the femoral resection accuracy, patient-reported outcome measures (PROMs), and component alignment were different from an experienced (E) surgeon. This prospective cohort study analyzed the IE surgeon’s first 30 TKAs, all performed with KA, and 30 consecutive KA TKAs performed by an E surgeon. The resection accuracy or deviation was the calipered thickness of the distal and posterior medial and lateral femoral resections minus the planned resection thickness, which was the thickness of the corresponding condyle of the femoral component, minus 2 mm for cartilage wear, and 1 mm for the kerf of the blade. Independent observers recorded the femoral resection thickness, operative times, PROMs, and alignment. For each femoral resection, the deviation between three groups of patients containing ten consecutive KA TKAs, was either insignificant (*p* = 0.695 to 1.000) or within the 0.5 mm resolution of the caliper, which indicated no learning curve. More than three groups were needed to determine the learning curve for the operative time; however, the IE surgeon’s procedure dropped to 77 min for the last 10 patients, which was 20 min longer than the E surgeon. The resection deviations of the IE and E surgeon were comparable, except for the posterolateral femoral resection, which the IE surgeon under-resected by a mean of −0.8 mm (*p* < 0.0001). At a mean follow-up of 9 and 17 months, the Forgotten Joint Score, Oxford Knee Score, KOOS, and the alignment of the components and limbs were not different between the IE and E surgeon (*p* ≥ 0.6994). A surgeon that switches to unrestricted KA with manual instruments can determine their learning curve by computing the deviation of the distal and posterior femoral resections from the planned resection. Based on the present study, an IE surgeon could have resection accuracy, post-operative patient outcomes, and component alignment comparable to an E surgeon.

## 1. Introduction

As up to 20% of patients after mechanically aligned (MA) total knee arthroplasty (TKA) are not satisfied with their postoperative knee function, alternative alignment options have gained interest with orthopedic surgeons [1]. Kinematically aligned (KA) TKA has demonstrated better patient outcomes in randomized trials, fewer complications, good implant survivorship, and biomechanics closer to native than MA TKA [2,3,4,5,6,7,8,9]. Motivated by these improvements in outcomes and biomechanics, surgeons considering a switch to KA with manual instruments are interested in knowing how many cases they need to perform to complete the learning curve.

Two metrics for assessing the learning curve are the accuracy of performing the femoral resections and the operative time. Correct distal and posterior femoral resections set the femoral component coincident with the patient’s pre-arthritic joint lines. Resection errors resulting in 1- and 2- degree deviations from the femoral and tibial joint lines cause high medial and lateral tibial compartment forces relative to the native knee [10,11,12,13]. The surgeon determines the accuracy, or deviation from the planned resection, by measuring the thickness of the distal and posterior medial and lateral femoral resections with a caliper and subtracting the thickness of the corresponding condyle of the femoral component, 2 mm for cartilage wear, and 1 mm for the kerf of the blade [14,15]. In addition, operative time concerns the transitioning surgeon striving for surgical efficiency. Statistically, a surgeon’s learning curve is considered complete when the deviation of each femoral resection from the planned resection and the operative time do not change between groups of patients, each containing ten consecutive cases [16].

The surgeon is interested in knowing whether inexperience in KA could adversely affect postoperative patient-reported outcome measures (PROMs), component alignment, and complications relative to an experienced surgeon. For example, the Forgotten Joint Score (FJS), Oxford Knee Score (OKS), and Knee injury and Osteoarthritis Outcome Score (KOOS) enable patients to report their level of satisfaction and function. In addition, the KA target for component alignment is to restore the patient’s pre-arthritic joint lines, which leads to, according to mechanical alignment (MA) criteria, a ‘varus outlier’ placement of the tibial component [17]. Hence, knowing that the range and mean difference in the component alignment is comparable to an E surgeon could ease concerns.

A prior study of less experienced surgeons showed the accuracy of resecting the femur using unrestricted KA and manual instruments was comparable to or better than robotic-arm surgery [15]. However, it did not report the learning curve and outcomes for a knee arthroplasty fellowship-trained surgeon that is inexperienced (IE) in KA and starting orthopedic practice. Accordingly, the present study reports the number of cases required to achieve accurate femoral resections and a consistent operating time. It also determines whether the IE surgeon’s accuracy, postoperative patient-reported outcome measures (PROMs), and component alignment are different from an experienced (E) surgeon.

## 2. Materials and Methods

An institutional review board approved this retrospective study (IRB 00060819) of deidentified prospectively collected data. In November 2019, a surgeon who completed a one-year arthroplasty fellowship that only taught MA TKA joined a clinic-based practice and became a surgeon’s partner, experienced in performing unrestricted KA TKA with manual instruments (i.e., >4000 cases). By reading available literature, attending a cadaver course, and observing the E surgeon operate, the IE surgeon performed all primary TKAs with KA and without excluding patients with severe varus, valgus, and flexion deformities using a previously described technique [18]. Excluded were patients with prior intra-articular fracture, bone loss from avascular necrosis, and septic arthritis. All patients that underwent primary TKA during the study period were operated on consecutively by the IE and E surgeon in the same hospital facility and signed a consent for the FDA-approved use of KA, which was the only alignment technique offered by the two surgeons.

### 2.1. Surgical Technique

**Figure 1:**
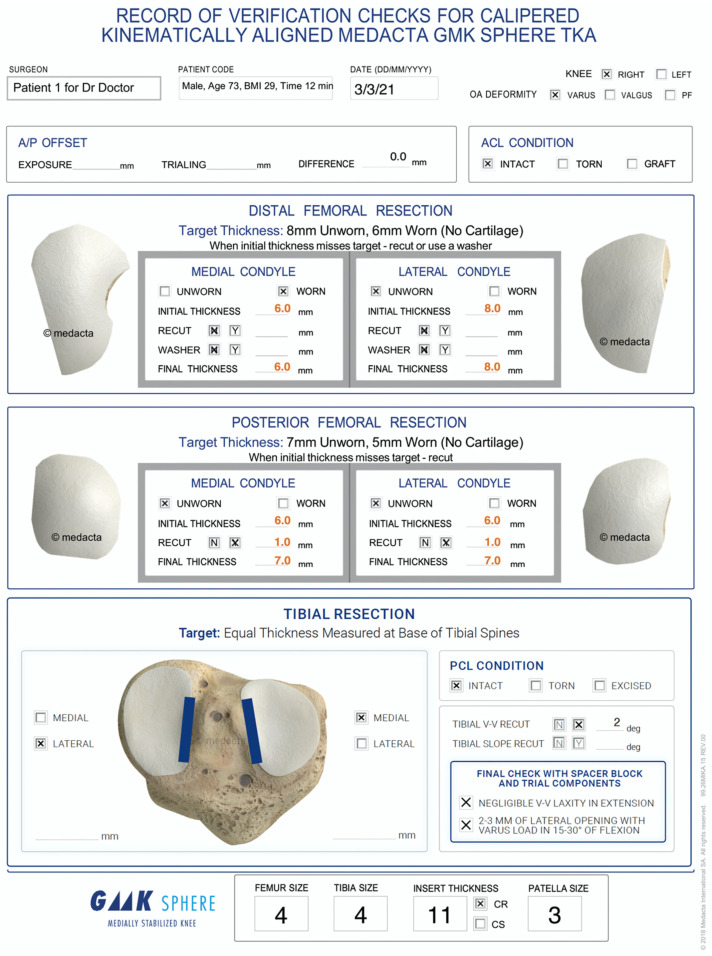
The image shows the intraoperative verification worksheet of a patient including entries for patient number, surgeon name, sex, age, BMI, time to complete corrected femoral cuts, date of surgery, right or left knee, type of primary deformity (varus, valgus, or patellofemoral), condition of ACL and PCL, target thickness of femoral resections, initial and corrected caliper-measured thickness of each femoral resection.

For the first thirty KA TKAs, an assistant intraoperatively recorded data on a verification worksheet (Figure 1). The worksheet provided a deidentified patient number, age, BMI, sex, date of surgery, right or left knee, type of primary deformity (varus, valgus, or patellofemoral), condition of ACL, the resection thickness of the distal medial, distal lateral, posterior medial, and posterior lateral femoral resections measured with a caliper to a resolution of ±0.5 mm, and the planned resection thickness, which was the thickness of the corresponding condyle of the femoral component, minus 2 mm for cartilage wear, and 1 mm for the kerf of the blade (Table 1).

The following steps describe the method for setting the femoral component’s varus-valgus (V-V) orientation and proximal-distal position coincident to the patient’s pre-arthritic distal femoral joint line with manual instruments. The basis for adjusting the target thickness for each femoral resection is knowing that full-thickness cartilage loss closely approximates 2 mm and that bone wear is negligible at 0° and 90° flexion in the Grade III and IV Kellgren–Lawrence osteoarthritic knee [19]. With the exposed knee at 90° of flexion, the pattern of cartilage wear is examined on the femur. Partially worn cartilage is removed to subchondral bone with a ring curette. The planned thickness for each resection is the thickness of the condyle of the femoral component, minus 2 mm for worn cartilage, and 1 mm for the thickness of the kerf or the saw cut. The distal resection is performed with a series of offset guides chosen to compensate for cartilage loss. The thickness of each distal femoral resection is measured with a caliper with a resolution of 0.5 mm and recorded on the verification worksheet (Figure 1). An under-resection of 1 mm or more is corrected by removing additional bone by (1) redirecting the saw blade through the cutting block, (2) using a 1- to 2-mm recut guide, or (3) free-handing the cut until within ±0.5 mm of the femoral target.

**Figure 2:**
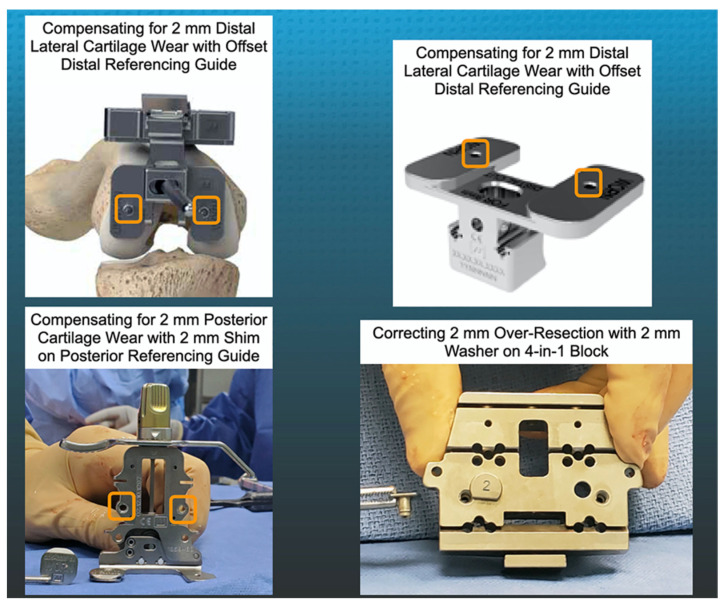
The composite of images shows the manual instruments used to make the distal and posterior femoral resections. The features include an offset distal referencing guide with two holes (orange squares) for compression screws (**upper left** and **right**), a posterior referencing guide set at 0° with small, medium, and large width posterior feet with two holes (orange squares) for compression screws and removable shims to compensate for 2 mm of distal and posterior cartilage wear (**lower left**), and a washer, available in 1 and 2 mm (shown) thicknesses, to correct for an over-resection of a distal femoral condyle (**lower right**).

The following steps describe the method for setting the femoral component’s internal–external (I-E) orientation and anterior–posterior (A-P) position coincident to the patient’s pre-arthritic posterior femoral joint line with manual instruments. The thickness of the cartilage in the posterior condyles is checked with a knife. When the cartilage is intact, the posterior referencing guide is set at 0° of rotation. When the cartilage is worn, a 2 mm shim is inserted between the foot of the referencing guide and the posterior condyle (Figure 2). Next, the correct size 4-in-1 cutting block is selected. When a distal resection was over-resected by 1- or 2-mm, a 1- or 2-mm washer was placed on the 4-in-1 cutting block. The washer displaces the 4-in-1 cutting block distally, which creates shallow anterior and posterior chamfer cuts and the gap from the distal femoral resection is filled with bone cement. The posterior femur is resected and the thickness is measured with a caliper. When a 1 mm or more under-resection is detected, the surgeon rotates the 4-in-1 chamfer block anterior about the fixation pin in the contralateral femoral condyle to expose enough bone to enable an additional resection that matches the thickness of the deviation. When a 1 mm or more over-resection is detected, the surgeon rotates the 4-in-1 chamfer block posterior about the fixation pin in the contralateral femoral condyle to create a gap that matches the thickness of the deviation, which is fillable with bone cement. Lastly, the anterior and posterior chamfers and anterior femur are resected.

The surgeon follows six options in a decision-tree to set the V-V orientation and posterior slope of the tibial component with the goal of restoring the patient’s pre-arthritic tibial joint line and limb alignment and balancing the knee. In the KA technique, the medial and lateral tibial resection thickness does not determine whether the varus–valgus orientation of the proximal tibial resection is correct. Instead, the varus–valgus orientation of the proximal tibial resection is adjusted, working in 1°–2° increments until a manual varus–valgus laxity assessment with the spacer block and trial components in extension show negligible medial and lateral lift-off. The method for visually selecting the posterior slope is to set an angel wing, inserted through the tibial guide’s medial slot, parallel to the slope of the medial tibial plateau [20].

### 2.2. Outcome Measures

The accuracy was the deviation of the distal and posterior medial and lateral femoral resections from the planned resection. Operative time was measured from skin incision to application of sterile dressing. At a minimum of 6 months after surgery, patients filled out the Forgotten Joint Score (FJS; 100 best, 0 worst), Oxford Knee Score (OKS; 48 best, 0 worst), and KOOS Jr. (100 best, 0 worst) questionnaires. Postoperative complications and their respective treatments were recorded.

Component positioning and limb alignment were measured on anteroposterior (AP), rotationally controlled, non-weight bearing, long-leg computed tomography (CT) scanogram of both limbs obtained on the day of hospital discharge [21]. An independent observer used image analysis software (Horos, v3.3.6, Annapolis, MD, USA) to measure the distal lateral femoral angle (DLFA), proximal medial tibial angle (PMTA), hip–knee–ankle angle (HKAA), flexion of the femoral component, and posterior slope of the tibial component [14,20,21,22]. To quantify repeatability and reproducibility, three observers independently performed all radiographic measurements three times, with at least 24 h between trials from the same five randomly selected patients from the IE and E surgeon (10 TKAs). The intraclass correlation coefficients (ICCs) were computed for each radiographic measurement with use of a two-factor ANOVA, where the two factors—modeled as random effects—were observed at 3 levels and patients at 10 levels. The resulting variance components for observer, patient, and error were used to compute the intraobserver pooled SDs and ICCs and interobserver ICCs. An ICC value of >0.9 indicates excellent agreement, and 0.75–0.90 indicates good agreement [23]. The pooled SD and ICC values for repeatability (i.e., intraobserver) and ICC values for reproducibility (i.e., interobserver) of the measurement HKAA (1.2°, 0.95, and 0.93), DLFA (1.7°, 0.95, and 0.95), PMTA (1.8°, 0.91, and 0.89), flexion of the FC (2.8°, 0.87, and 0.81), and slope of the TC (2.4°, 0.90, and 0.89) indicated good or excellent agreement.

### 2.3. Statistical Analysis

A sample size calculation determined the number of consecutive patients allocated into groups to compute the learning curve for the resection accuracy of the IE surgeon. One assumption was that a 1.0 mm deviation from the planned resection thickness was of clinical importance. In addition, the 1.0 mm deviation was conservative because a 2 mm increase in the joint line level stiffens the knee by doubling the medial and lateral tibial compartment forces from native [13]. Other assumptions were a Type I error (alpha) of 0.05, a power (1-beta) of 0.95, and a 0.5 mm standard deviation from the resection target that a prior study that analyzed femoral resection accuracy reported [15]. Hence, the sample size calculation of ten patients per group (=30 patients in three groups) to detect a 1 mm mean deviation between groups showed the present study was sufficiently powered to reduce the risk of a Type-II error.

An ANOVA analyzed the IE surgeons’ learning curve (JMP^®^ Pro 16.0.0, www.jmp.com, SAS, Cary, NC, USA). The independent variable was the patient group with three levels (1–10 TKAs, 11–20 TKAs, and 21–30 TKAs), and the dependent variables were the deviation of each femoral resection and operative time. A post-hoc Tukey’s test determined the number of cases (i.e., group) at which each femoral resection deviation and operative time did not change. The Fisher’s Exact Test for categorical variables, Student’s T-test for normally distributed data, and the Wilcoxon Rank-Sum Test for non-normally distributed data determined differences in each femoral resection deviation, component alignment, and PROMs between the IE and E surgeon. Statistical significance was set at *p* < 0.05.

## 3. Results

Pre-operatively, there were no differences in patient characteristics, knee conditions, and function scores between the patients treated by the IE and E surgeons, except the OKS was six points lower for the IE surgeon’s patients (Table 1).

**Figure 3:**
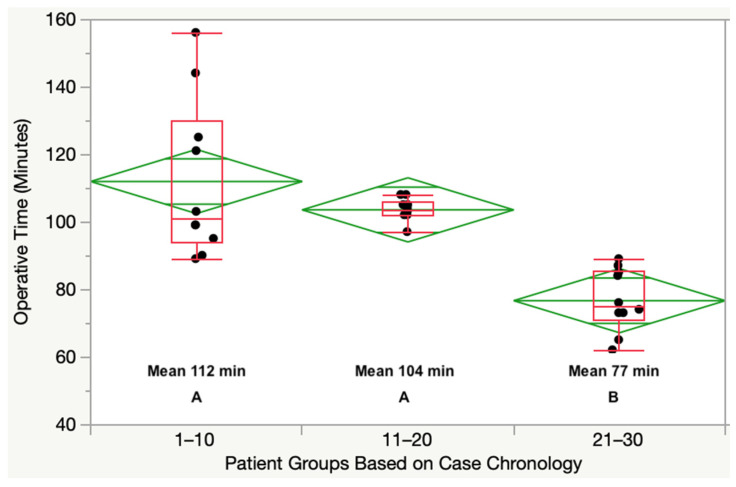
Boxplot shows the improvement of the IE surgeon’s mean operative time from 112 min for the first 10 cases to 77 min for the last ten cases. Patient groups not connected by the same letter are significantly different (*p* = 0.0003 to <0.0001).

The IE surgeon did not have a learning curve performing the distal and posterior medial and lateral femoral resections. The mean deviation from the planned resection did not change between the three patient groups of ten patients (Table 2). The learning curve for the operative time was indeterminate; however, the IE surgeon’s procedure dropped from 104 min for patients in the 11–20 group to 77 min for patients in the 21–30 group (Figure 3).

The accuracy of each femoral resection was not different between the inexperienced and experienced surgeons, except for the posterior lateral femoral resection (Table 3).

The mean time between surgery and the final PROM evaluation was significantly shorter for the IE surgeon (9 months) relative to the E surgeon (17 months). However, there were no differences in FJS, OKS, and KOOS between the IE and E surgeons (Table 4).

The four radiologic measures of component and limb alignment were not significantly different between the IE and E surgeons (Table 5). The IE and E surgeons’ patients reported no complications within six weeks of surgery.

## 4. Discussions

The first important finding of the present study was that an IE surgeon trained in MA TKA that switched to unrestricted KA TKA with manual instruments after an arthroplasty fellowship required no learning curve to perform the distal and posterior femoral resections accurately. The second was that the operative time learning curve remained indeterminate, although time improved from 114 min for the first ten cases to 71 min for the last ten cases. The third was that the IE surgeon achieved the same femoral resection accuracy (except for posterolateral), patient-reported outcome scores, and component and limb alignments as an E surgeon, but required more operative time.

One explanation for the IE surgeon’s accuracy in resecting the femur and the lack of a learning curve is that most surgeons skillfully use a caliper to fine-tune the patella resection thickness when resurfacing the patella, which is an essential part of caliper-verified KA TKA. A second is that planning the resection thicknesses requires a simple computation, enabling the surgeon to fix manual distal and posterior referencing guides to the articular surface and cut the femur to restore the pre-arthritic femoral joint lines accurately. Directly registering the manual cutting guides to the femoral articular surface eliminates the stacked errors intrinsic to robotic, navigation, and patient-specific instrumentation systems caused by converting preoperative images into a 3D model, virtually planning resection planes, and registering the navigation or robotic unit to the patient [24,25]. The cause of the IE surgeon’s −0.8 mm under-resection of the posterolateral femur was an interposed remnant of the lateral meniscus between the posterior referencing guide and femoral condyle, which is easy to prevent with better exposure. The value of the caliper verification is that it detected a deviation from the planned posterolateral resection thickness before performing the anterior and posterior chamfer and anterior femoral resections. The IE surgeon corrected the initial resection by rotating the 4-in-1 chamfer block anterior about the fixation pin in the contralateral femoral condyle to expose enough bone to enable an additional resection that matched the thickness of the deviation. Hence, the IE surgeon’s deviations from the planned femoral resections were comparable to the E surgeon’s and comparable to or more accurate than robotic arm instrumentation [8,26,27].

The IE surgeon took a mean time of 112 min for the first 10 TKAs compared to 77 for the 21–30th TKAs, which is an economic improvement of 35 min that freed up the operating room. An explanation for the IE surgeon’s rapid reduction in operative time performing unsupervised TKA for the first time after an arthroplasty fellowship was the acquisition of decision-making confidence, finely-tuned surgical skills, and familiarity with the surgical steps that are different from MA. Compared to the mean operative time of 116 min for MA TKA by four fellowship-trained arthroplasty surgeons at an urban, academic institution, the IE surgeon was more efficient [28]. In addition, a senior surgeon using an electronic sensor took 109 min to balance an MA TKA; however, in 38 min less time, the caliper-verified KA technique, by default, restored the native medial and lateral tibial compartment forces without ligament release [12,13]. Hence, surgeons switching to KA might find these comparisons comforting.

Any surgeon starting practice naturally harbors uncertainties about whether their surgical outcomes match the standards established by more experienced colleagues. The IE surgeon’s patients reported similar FJS, OKS, and KOOS to the E surgeon because the range and mean values of the sagittal and coronal component alignment and the HKAA matched the E surgeon. However, the follow-up time of the IE surgeon’s patients of 9 months was shorter than a year, and shorter than the 17-month follow-up of the E surgeon, because it took 15 more months to perform the 30 KA TKAs. This difference in the follow-up could influence the comparison because PROMs improve with statistical and clinical significance between 4 months and 1 year, as improvements from a 1-year to 2-year follow-up do not reach a minimal clinically important difference (MCID) [29]. However, because the PROMs were similar between the two surgeons, the conclusion that an IE surgeon achieves comparable results to an E surgeon is justified. The comparable PROMs can be attributed to both surgeons using the same principles to cut the femur, to the planned resection thicknesses, and the tibia, to establish a tight rectangular extension space and restore the slope of the medial tibial joint line [14,20,21].

There are limitations to generalizing the results from the present study which the reader should understand. First and foremost, the femoral resection and operative time learning curves represent the experience of only the IE surgeon that learned the unrestricted caliper-verified KA technique through extensive reading, performing TKAs in cadaveric specimens, and assisting the E surgeon. Hence, the learning curves reported in the present study might differ from other IE surgeons that do not have the same educational dedication. Second, the deviations from the planned resection thickness were applied to the system of manual instruments designed for caliper-verified KA that used compression screws to securely fix the distal and posterior referencing guides to the femur and may not be generalizable to other systems. Third, the high PROMs were achieved using manual instrumentation that did not always resect the femur to the planned thickness. However, the IE and E surgeons corrected over- and under-resection deviations from the planned resection thickness detected with a caliper, resulting in a more accurate component implantation. These validation checks and corrective steps could benefit robotic, navigational, and patient-specific instrumentation, as they have larger resection deviations than those of the present study [24,25,27,30]. Finally, a gate analysis could have provided an additional clinical outcome measure to compare the three patient groups of the IE surgeon and the patients between the IE and E surgeon [31].

## 5. Conclusions

The present study fills several gaps in knowledge that might interest an IE surgeon that switches to unrestricted caliper-verified KA TKA performed with manual instruments. First, they can use the described methodology of structuring three or more patient groups based on case chronology and determine their learning curves by computing the deviation of the distal and posterior femoral resections from the planned resection thickness and recording the operative time. Finally, they can compare their resection accuracy, operative time, PROMs, and component alignment to those of the E surgeon.

## Figures and Tables

**Table 1:** Shows pre-operative characteristics, knee conditions, and function scores for the 30 patients treated by inexperienced and experienced surgeons and significant differences.

		Inexperienced Surgeon	Experienced Surgeon (>4000 KA TKA)	Significance
Number of Days to Perform 30 Consecutive KA TKAs		441	17	
**Patient Characteristics**				
Age (years)	Mean ± SD	68 ± 8.5	69 ± 7.6	NS, *p* = 0.4845 *
Body Mass Index (kg/m^2^)	Mean ± SD	31 ± 4.5	30 ± 6.2	NS, *p* = 0.4675 *
Knee Sidedness		18 right, 12 left	11 right, 19 left	NS, *p* = 0.1205 ^#^
Sex		14 male, 16 female	14 male, 16 female	NS, *p* = 1.0000 ^#^
OA Deformity		21 varus, 8 valgus, 1 patellofemoral	22 varus, 8 valgus	NS, *p* = 0.6235 ^#^
ACL Condition		23 intact, 5 torn, 2 reconstructed	21 intact, 9 torn	NS, *p* = 0.2411 ^#^
**Preoperative Function Scores**				
Oxford Knee Score (OKS) (48 best, 0 worst)	Mean ± SD	18 ± 7.5	24 ± 9.8	*p* = 0.0146 *
Knee Injury and Osteoarthritis Outcome Score (KOOS) (100 best, 0 worst)	Mean ± SD	41 ± 17.6	49 ± 17.3	NS, *p* = 0.0854 *

* Student’s *T*-test, ^#^ Fisher’s Exact Test, Standard Deviation (SD), Non-Significant (NS).

**Table 2:** Shows no difference in the mean deviation of each femoral resection from the planned resection across the three patient groups (ANOVA), which indicated the IE surgeon did not have a learning curve.

		Groups of Ten Patients Treated by Inexperienced Surgeon			
		1–10 KA TKAs	11–20 KA TKAs	21–30 KA TKAs	Significance
Distal Medial Femoral Deviation from Planned Resection (mm)	Mean ± SD	−0.2 ± 0.3	−0.2 ± 0.5	−0.2 ± 0.2	NS, *p* = 0.9403
Distal Lateral Femoral Deviation from Planned Resection (mm)	Mean ± SD	0.3 ± 0.5	−0.3 ± 0.5	−0.3 ± 0.5	NS, *p* = 0.0534
Posterior Medial Femoral Deviation from Planned Resection (mm)	Mean ± SD	−0.4 ± 0.9	−0.4 ± 0.5	−0.3 ± 0.6	NS, *p* = 0.9830
Posterior Lateral Femoral Deviation from Planned Resection (mm)	Mean ± SD	−1.2 ± 1.0	−0.5 ± 0.6	−0.8 ± 1.2	NS, *p* = 0.2449

Standard Deviation (SD), Non-Significant (NS).

**Table 3:** Shows the accuracy of each femoral resection, measured as the mean deviation from the planned resection, which was not different between the inexperienced and experienced surgeon, except for the posterior lateral femoral resection (Student’s *T*-test *).

		Surgeon’s Level of Experience		
		Inexperienced	Experienced (>4000 KA TKA)	*Significance*
Distal Medial Femoral Deviation from the Planned Resection (mm)	Mean ± SD	−0.2 ± 0.4	−0.0 ± 0.5	NS, *p* = 0.2327 *
Distal Lateral Femoral Deviation from the Planned Resection (mm)	Mean ± SD	−0.1 ± 0.5	−0.0 ± 0.5	NS, *p* = 0.7048 *
Posterior Medial Femoral Deviation from the Planned Resection (mm)	Mean ± SD	−0.3 ± 0.7	−0.1 ± 1.0	NS, *p* = 0.3427 *
Posterior Lateral Femoral Deviation from the Planned Resection (mm)	Mean ± SD	−0.8 ± 1.0	0.1 ± 0.6	*p* < 0.0001 *

Standard Deviation (SD), Non-Significant (NS).

**Table 4:** Shows the number of months from surgery to final follow-up and that the Forgotten Joint Score, Oxford Knee Score, and KOOS were not significantly different between the inexperienced and experienced surgeons ().

		Surgeon’s Level of Experience		
		Inexperienced	Experienced (>4000 KA TKA)	Significance
Follow-up (months from surgery)	Mean ± SD	9 ± 3	17 ± 5	*p* < 0.0001 *
Forgotten Joint Score (FJS) (100 best, 0 worst)	Median, IQR	88 (71 to 92)	81 (56 to 100)	NS, *p* = 0.6994 ^#^
Oxford Knee Score (OKS) (48 best, 0 worst)	Median, IQR	43 (40 to 46)	45 (39 to 47)	NS, *p* = 0.8879 ^#^
Knee Injury and Osteoarthritis Outcome Score (KOOS) (100 best, 0 worst)	Median, IQR	80 (73 to 92)	79 (62 to 100)	NS, *p* = 0.9145 ^#^

* Student’s *T*-test, ^#^ Wilcoxon Rank-Sum Test, Standard deviation (SD), interquartile range (IQR), Non-Significant (NS).

**Table 5:** Shows the component and limb alignment were not significantly different between the inexperienced and experienced surgeons (Student’s *T*-test).

		Surgeon’s Level of Experience		
		Inexperienced	Experienced (>4000 KA TKA)	*Significance*
Hip–Knee–Ankle Angle (HKAA) (°)	Range	−5 (valgus) to 10 (varus)	−6 (valgus) to 6 (varus)	
	Mean ± SD	2 ± 3.1	1 ± 3.1	NS, *p* = 0.4027
Distal–Lateral–Femoral Angle (DLFA) (°)	Range	82 (valgus) to 91 (varus)	83 (valgus) to 93 (varus)	
	Mean ± SD	87 ± 2.4	87 ± 2.7	NS, *p* = 0.7521
Proximal–Medial–Tibial Angle (PMTA) (°)	Range	82 (varus) to 89 (valgus)	79 (varus) to 95 (valgus)	
	Mean ± SD	86 ± 2.0	86 ± 2.7	NS, *p* = 0.2091
Flexion of the Femoral Component (°)	Range	−3 (extension) to 10 (flexion)	−5 (extension) to 7 (flexion)	
	Mean ± SD	4 ± 2.8	3 ± 3.4	NS, *p* = 0.1876
Slope of the Tibial Component (°)	Range	−2 (extension) to 10 (flexion)	−4 (extension) to 12 (flexion)	
	Mean ± SD	4 ± 3.3	4 ± 3.8	NS, *p* = 0.6864

Standard Deviation (SD), Non-Significant (NS).
